# Bis[2-(amino­meth­yl)pyridine-κ^2^ *N*,*N*′]bis­(thio­cyanato-κ*N*)copper(II)

**DOI:** 10.1107/S1600536809013427

**Published:** 2009-04-18

**Authors:** Nirmal Kumar Karan, Kai-Ting Chan, Hon Man Lee

**Affiliations:** a National Changhua University of Education, Department of Chemistry, Changhua, Taiwan 50058

## Abstract

In the title complex, [Cu(NCS)_2_(C_6_H_8_N_2_)_2_], the Cu^II^ atom, lying on an inversion center, adopts a Jahn–Teller distorted octahedral CuN_6_ coordination geometry. The two bidentate 2-amino­methyl­pyridine ligands are coordinated in a *trans* fashion, while the two thio­cyanate ligands are at the axial positions and coordinate to the Cu atom in a bent mode with a C—N—Cu angle of 127.49 (10)°. Inter­molecular N—H⋯N and N—H⋯S hydrogen bonds link the copper complex mol­ecules into an infinite two-dimensional network.

## Related literature

For six-coordinate *trans*-dithio­cyanato Cu(II) complexes similar to the title complex, see: Gary *et al.* (2004 ▶); Ferrer *et al.* (1992 ▶); Gorji *et al.* (2001 ▶); Kozlowski & Hodgson (1975 ▶); Li & Zhang (2004 ▶).
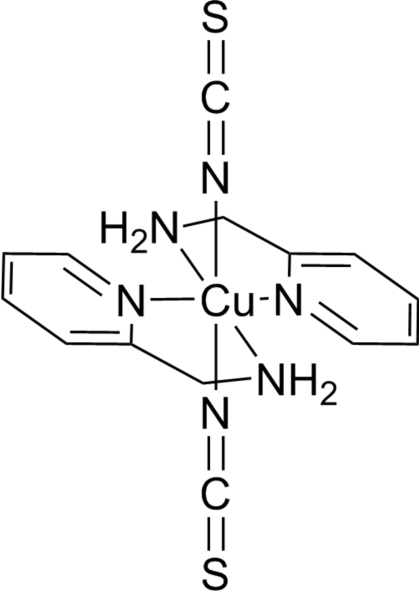


## Experimental

### 

#### Crystal data

[Cu(NCS)_2_(C_6_H_8_N_2_)_2_]
*M* *_r_* = 395.99
Monoclinic, 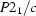

*a* = 9.1023 (4) Å
*b* = 9.1740 (4) Å
*c* = 9.6895 (4) Å
β = 91.872 (3)°
*V* = 808.69 (6) Å^3^
*Z* = 2
Mo *K*α radiation
μ = 1.62 mm^−1^
*T* = 150 K
0.46 × 0.38 × 0.31 mm

#### Data collection

Bruker SMART APEXII diffractometer
Absorption correction: multi-scan (*SADABS*; Sheldrick, 2003 ▶) *T*_min_ = 0.497, *T*_max_ = 0.603
11419 measured reflections
2086 independent reflections
1776 reflections with *I* > 2σ(*I*)
*R*_int_ = 0.031

#### Refinement

*R*[*F* ^2^ > 2σ(*F* ^2^)] = 0.023
*wR*(*F* ^2^) = 0.063
*S* = 1.13
2086 reflections
106 parameters
H-atom parameters constrained
Δρ_max_ = 0.35 e Å^−3^
Δρ_min_ = −0.40 e Å^−3^

### 

Data collection: *APEX2* (Bruker, 2004 ▶); cell refinement: *SAINT* (Bruker, 2004 ▶); data reduction: *SAINT*; program(s) used to solve structure: *SHELXS97* (Sheldrick, 2008 ▶); program(s) used to refine structure: *SHELXL97* (Sheldrick, 2008 ▶); molecular graphics: *SHELXTL* (Sheldrick, 2008 ▶); software used to prepare material for publication: *SHELXTL*.

## Supplementary Material

Crystal structure: contains datablocks I, global. DOI: 10.1107/S1600536809013427/pv2151sup1.cif

Structure factors: contains datablocks I. DOI: 10.1107/S1600536809013427/pv2151Isup2.hkl

Additional supplementary materials:  crystallographic information; 3D view; checkCIF report

## Figures and Tables

**Table 1:** Hydrogen-bond geometry (Å, °)

*D*—H⋯*A*	*D*—H	H⋯*A*	*D*⋯*A*	*D*—H⋯*A*
N2—H2*A*⋯N3^i^	0.92	2.27	3.0717 (19)	145
N2—H2*B*⋯S1^ii^	0.92	2.60	3.4628 (13)	156

Symmetry codes: (i) 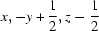; (ii) 
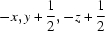
.

**Fig. 1.**
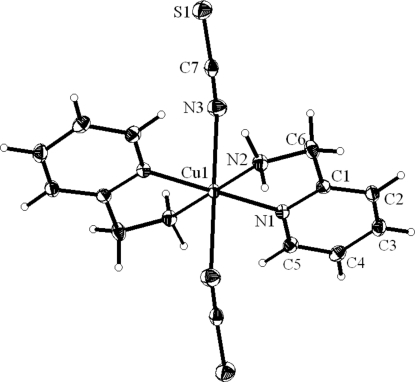
The structure of the title complex, showing 50% displacement ellipsoids for non-H atoms. The H atoms are dipicted by circles of an arbitrary radius. The unlabelled atoms are related to the labelled ones by -x, 1 - y, 1 - z.

**Fig. 2.**
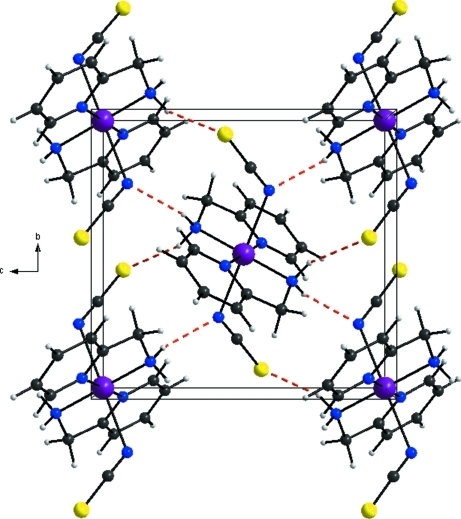
A packing diagram of the title compound along the a axis. Hyrogen bonds are shown as dashed lines.

## supplementary crystallographic information

### Comment

The tilte complex was readily obtained from the reaction between copper(II) acetate, 2-aminomethylpyridine, and sodium thiocyanate. The complex consists of two *trans* bidentate ligands and two *trans* thiocyanate ligands (Fig. 1). It exhibits an octahedron coordination geometry at the Cu atom which is located on the inversion center. The bent coordination of thiocyanate results in the C5—N1—Cu1 bond angle of 127.49 (10)°. The pyridine ring is twisted from the equatorial plane defined by the Cu and the four N atoms; the interplanar angle is 17.86 (8)°. Intermolecular H-bonds of the type N—H···N and N—H···S exist, linking the complex into a into a two-dimensional hydrogen bonded network (Table 1).

Six-coordinate *trans*-dithiocyanato Cu(II) complexes similar to the title complex have been reported in the literature (Gary *et al.*, 2004; Ferrer *et al.*, 1992; Gorji *et al.*, 2001; Kozlowski & Hodgson, 1975; Li & Zhang, 2004).

### Experimental

To a methanolic solution (10 ml) of Cu(O_2_CCH_3_)_2_.H_2_O (1.0 mmol, 0.199 g), a methanolic solution (10.0 ml) of 2-aminomethylpyridine (2.0 mmol, 0.207 ml) was added dropwise with stirring. Then to this mixture of solution, NaSCN (2.0 mmol, 0.162 g) in methanol (5.0 ml) was added and the mixture was stirred for 5 min. The solution was kept undisturbed. Blue crystals suitable for *X*-ray crystallography were obtained after one week by slow evaporation of the solvent.

### Refinement

All the H atoms were positioned geometrically and refined as riding atoms, with N—H = 0.92, C_aryl_—H = 0.95, C_methylene_—H = 0.99 Å while *U*_iso_(H) = 1.2*U*_eq_(C or N) for all H atoms.

### Crystal data

**Table:**

[Cu(NCS)_2_(C_6_H_8_N_2_)_2_]	*F*(000) = 406
*M**_r_* = 395.99	*D*_x_ = 1.626 Mg m^−^^3^
Monoclinic, *P*2_1_/*c*	Mo *K*α radiation, λ = 0.71073 Å
Hall symbol: -P 2ybc	Cell parameters from 3360 reflections
*a* = 9.1023 (4) Å	θ = 3.1–28.0°
*b* = 9.1740 (4) Å	µ = 1.62 mm^−^^1^
*c* = 9.6895 (4) Å	*T* = 150 K
β = 91.872 (3)°	Plate, blue
*V* = 808.69 (6) Å^3^	0.46 × 0.38 × 0.31 mm
*Z* = 2	

### Data collection

**Table:**

Bruker SMART APEXII diffractometer	2086 independent reflections
Radiation source: fine-focus sealed tube	1776 reflections with *I* > 2σ
graphite	*R*_int_ = 0.031
ω scans	θ_max_ = 28.7°, θ_min_ = 2.2°
Absorption correction: multi-scan (*SADABS*; Sheldrick, 2003)	*h* = −12→12
*T*_min_ = 0.497, *T*_max_ = 0.603	*k* = −12→12
11419 measured reflections	*l* = −13→12

### Refinement

**Table:**

Refinement on *F*^2^	Primary atom site location: structure-invariant direct methods
Least-squares matrix: full	Secondary atom site location: difference Fourier map
*R*[*F*^2^ > 2σ(*F*^2^)] = 0.023	Hydrogen site location: inferred from neighbouring sites
*wR*(*F*^2^) = 0.063	H-atom parameters constrained
*S* = 1.13	*w* = 1/[σ^2^(*F*_o_^2^) + (0.0308*P*)^2^ + 0.2097*P*] where *P* = (*F*_o_^2^ + 2*F*_c_^2^)/3
2086 reflections	(Δ/σ)_max_ < 0.001
106 parameters	Δρ_max_ = 0.35 e Å^−^^3^
0 restraints	Δρ_min_ = −0.40 e Å^−^^3^

### Special details

**Table:**

Geometry. All e.s.d.'s (except the e.s.d. in the dihedral angle between two l.s. planes) are estimated using the full covariance matrix. The cell e.s.d.'s are taken into account individually in the estimation of e.s.d.'s in distances, angles and torsion angles; correlations between e.s.d.'s in cell parameters are only used when they are defined by crystal symmetry. An approximate (isotropic) treatment of cell e.s.d.'s is used for estimating e.s.d.'s involving l.s. planes.
Refinement. Refinement of *F*^2^ against ALL reflections. The weighted *R*-factor *wR* and goodness of fit *S* are based on *F*^2^, conventional *R*-factors *R* are based on *F*, with *F* set to zero for negative *F*^2^. The threshold expression of *F*^2^ > σ(*F*^2^) is used only for calculating *R*-factors(gt) *etc*. and is not relevant to the choice of reflections for refinement. *R*-factors based on *F*^2^ are statistically about twice as large as those based on *F*, and *R*- factors based on ALL data will be even larger.

### Fractional atomic coordinates and isotropic or equivalent isotropic displacement parameters (Å^2^)

**Table:**

	*x*	*y*	*z*	*U*_iso_*/*U*_eq_	
C1	0.27555 (16)	0.35413 (16)	0.48964 (15)	0.0151 (3)	
C2	0.41671 (16)	0.30420 (16)	0.52349 (17)	0.0187 (3)	
H2	0.4607	0.2302	0.4701	0.022*	
C3	0.49184 (16)	0.36421 (17)	0.63614 (17)	0.0199 (3)	
H3	0.5878	0.3311	0.6618	0.024*	
C4	0.42557 (17)	0.47314 (17)	0.71109 (17)	0.0190 (3)	
H4	0.4759	0.5170	0.7878	0.023*	
C5	0.28491 (17)	0.51688 (16)	0.67232 (16)	0.0170 (3)	
H5	0.2395	0.5917	0.7236	0.020*	
C6	0.18680 (17)	0.29582 (17)	0.36793 (16)	0.0188 (3)	
H6A	0.2513	0.2813	0.2888	0.023*	
H6B	0.1435	0.2005	0.3918	0.023*	
C7	−0.15576 (16)	0.17946 (16)	0.52438 (16)	0.0175 (3)	
Cu1	0.0000	0.5000	0.5000	0.01369 (8)	
N1	0.20991 (13)	0.45719 (14)	0.56437 (13)	0.0142 (2)	
N2	0.06857 (13)	0.39999 (14)	0.32960 (13)	0.0161 (3)	
H2A	−0.0087	0.3513	0.2869	0.019*	
H2B	0.1030	0.4680	0.2688	0.019*	
N3	−0.08726 (15)	0.26276 (15)	0.58955 (15)	0.0244 (3)	
S1	−0.25589 (5)	0.06047 (5)	0.43612 (4)	0.02601 (11)	

### Atomic displacement parameters (Å^2^)

**Table:**

	*U*^11^	*U*^22^	*U*^33^	*U*^12^	*U*^13^	*U*^23^
C1	0.0159 (7)	0.0142 (6)	0.0154 (7)	0.0005 (5)	0.0020 (5)	0.0008 (5)
C2	0.0166 (7)	0.0171 (7)	0.0225 (8)	0.0036 (6)	0.0032 (6)	0.0011 (6)
C3	0.0140 (7)	0.0215 (7)	0.0243 (8)	0.0029 (6)	0.0003 (6)	0.0069 (6)
C4	0.0167 (7)	0.0224 (8)	0.0177 (8)	−0.0027 (6)	−0.0030 (6)	0.0016 (6)
C5	0.0164 (7)	0.0187 (7)	0.0159 (7)	−0.0008 (6)	0.0001 (6)	−0.0009 (5)
C6	0.0187 (7)	0.0201 (7)	0.0176 (8)	0.0052 (6)	−0.0015 (6)	−0.0049 (6)
C7	0.0168 (7)	0.0183 (7)	0.0175 (8)	0.0037 (6)	0.0027 (6)	0.0057 (6)
Cu1	0.01194 (13)	0.01631 (13)	0.01273 (14)	0.00240 (9)	−0.00107 (9)	−0.00347 (9)
N1	0.0130 (6)	0.0152 (5)	0.0143 (6)	0.0009 (5)	0.0004 (5)	0.0000 (5)
N2	0.0154 (6)	0.0190 (6)	0.0140 (6)	0.0017 (5)	−0.0006 (5)	−0.0019 (5)
N3	0.0261 (7)	0.0213 (7)	0.0255 (8)	−0.0016 (6)	−0.0033 (6)	0.0040 (6)
S1	0.0235 (2)	0.0293 (2)	0.0251 (2)	−0.00253 (17)	−0.00159 (16)	−0.00652 (17)

### Geometric parameters (Å, °)

**Table:**

C1—N1	1.3430 (19)	C6—N2	1.4775 (18)
C1—C2	1.393 (2)	C6—H6A	0.9900
C1—C6	1.506 (2)	C6—H6B	0.9900
C2—C3	1.383 (2)	C7—N3	1.160 (2)
C2—H2	0.9500	C7—S1	1.6440 (16)
C3—C4	1.385 (2)	Cu1—N2	2.0062 (12)
C3—H3	0.9500	Cu1—N2^i^	2.0062 (12)
C4—C5	1.382 (2)	Cu1—N1	2.0288 (12)
C4—H4	0.9500	Cu1—N1^i^	2.0288 (12)
C5—N1	1.3466 (19)	N2—H2A	0.9200
C5—H5	0.9500	N2—H2B	0.9200
			
N1—C1—C2	121.85 (14)	C1—C6—H6B	109.8
N1—C1—C6	115.83 (12)	H6A—C6—H6B	108.2
C2—C1—C6	122.32 (13)	N3—C7—S1	178.26 (15)
C3—C2—C1	118.90 (14)	N2—Cu1—N2^i^	180.0
C3—C2—H2	120.5	N2—Cu1—N1	81.33 (5)
C1—C2—H2	120.5	N2^i^—Cu1—N1	98.67 (5)
C2—C3—C4	119.23 (14)	N2—Cu1—N1^i^	98.67 (5)
C2—C3—H3	120.4	N2^i^—Cu1—N1^i^	81.33 (5)
C4—C3—H3	120.4	N1—Cu1—N1^i^	180.0
C5—C4—C3	118.84 (14)	C1—N1—C5	118.81 (13)
C5—C4—H4	120.6	C1—N1—Cu1	113.68 (10)
C3—C4—H4	120.6	C5—N1—Cu1	127.49 (10)
N1—C5—C4	122.33 (14)	C6—N2—Cu1	109.43 (9)
N1—C5—H5	118.8	C6—N2—H2A	109.8
C4—C5—H5	118.8	Cu1—N2—H2A	109.8
N2—C6—C1	109.56 (12)	C6—N2—H2B	109.8
N2—C6—H6A	109.8	Cu1—N2—H2B	109.8
C1—C6—H6A	109.8	H2A—N2—H2B	108.2
N2—C6—H6B	109.8		
			
N1—C1—C2—C3	−0.9 (2)	C6—C1—N1—Cu1	3.52 (16)
C6—C1—C2—C3	179.38 (14)	C4—C5—N1—C1	−1.7 (2)
C1—C2—C3—C4	−0.7 (2)	C4—C5—N1—Cu1	176.34 (11)
C2—C3—C4—C5	1.1 (2)	N2—Cu1—N1—C1	−17.99 (10)
C3—C4—C5—N1	0.1 (2)	N2^i^—Cu1—N1—C1	162.01 (10)
N1—C1—C6—N2	19.63 (18)	N2—Cu1—N1—C5	163.84 (13)
C2—C1—C6—N2	−160.67 (14)	N2^i^—Cu1—N1—C5	−16.16 (13)
C2—C1—N1—C5	2.2 (2)	C1—C6—N2—Cu1	−33.03 (14)
C6—C1—N1—C5	−178.14 (13)	N1—Cu1—N2—C6	27.99 (10)
C2—C1—N1—Cu1	−176.18 (11)	N1^i^—Cu1—N2—C6	−152.01 (10)

Symmetry codes: (i) −*x*, −*y*+1, −*z*+1.

### Hydrogen-bond geometry (Å, °)

**Table:**

*D*—H···*A*	*D*—H	H···*A*	*D*···*A*	*D*—H···*A*
N2—H2A···N3^ii^	0.92	2.27	3.0717 (19)	145
N2—H2B···S1^iii^	0.92	2.60	3.4628 (13)	156

Symmetry codes: (ii) *x*, −*y*+1/2, *z*−1/2; (iii) −*x*, *y*+1/2, −*z*+1/2.
